# Delusional thinking and action binding in healthy individuals

**DOI:** 10.1038/s41598-021-97977-y

**Published:** 2021-09-23

**Authors:** Liyu Cao, Michael B. Steinborn, Barbara F. Haendel

**Affiliations:** 1 Department of Psychology and Behavioural Sciences, Zhejiang University, Tianmushan Road 148, Hangzhou, 310007 China; 2 Department of Psychology (III), Julius-Maximilians-Universität Würzburg, 97070 Würzburg, Germany

**Keywords:** Cognitive neuroscience, Psychology

## Abstract

Action binding is the effect that the perceived time of an action is shifted towards the action related feedback. A much larger action binding effect in schizophrenia compared to normal controls has been shown, which might be due to positive symptoms like delusions. Here we investigated the relationship between delusional thinking and action binding in healthy individuals, predicting a positive correlation between them. The action binding effect was evaluated by comparing the perceived time of a keypress between an operant (keypress triggering a sound) and a baseline condition (keypress alone), with a novel testing method that massively improved the precision of the subjective timing measurement. A positive correlation was found between the tendency of delusional thinking (measured by the 21-item Peters et al. delusions inventory) and action binding across participants after controlling for the effect of testing order between operant and baseline conditions. The results indicate that delusional thinking in particular influences action time perception and support the notion of a continuous distribution of schizotypal traits with normal controls at one end and clinical patients at the other end.

## Introduction

The perception of action time is heavily influenced by action related sensory feedback. Action binding demonstrates such an influence by showing that the perceived time of a keypress is shifted towards the time of a delayed sound triggered by the keypress^1^. Two testing conditions are needed to measure the action binding effect. In the baseline condition, voluntary keypresses are made without any auditory feedback, and the keypress time is reported. In the operant condition, everything is the same except that each voluntary keypress triggers a sound feedback with a short delay (typically 250 ms). The reported keypress time in the operant condition is delayed relative to the baseline condition. Action binding is often studied together with outcome binding, which refers to the effect that the perceived time of an action outcome (e.g. a sound) is shifted back towards the time of the outcome-triggering action. The two effects together are called intentional binding, with the idea behind that the voluntary intention of action execution causes the binding effects^1,2^. However, Wolpe and colleagues showed evidence that the two binding effects may be driven by different mechanisms^3^. When the two binding effects are discussed as a whole, the underlying cognitive mechanism is also debated. Alternative accounts to the intention explanation include the causality hypothesis^4^ (i.e. the knowledge of action causing its sensory feedback leads to the binding effects) and the cue integration idea^3,5^. The cue integration idea assumes that the binding effects are the results of a weighted integration of timing information from the action and the action outcome. Thus, it is not mutually exclusive with either the intention account or the causality account. In the present study, we focus on the action binding effect alone.

Interestingly, a much stronger action binding effect in schizophrenia has been shown compared to normal controls^6^. In particular, positive symptoms like delusions seem to contribute to the abnormal action binding in schizophrenia^7^. Delusions are false beliefs that contradict the reality held by the majority of the population (e.g. believing that people can communicate telepathically). The notion of ‘a psychosis continuum’ suggests that psychotic symptoms as found in clinical patients are also present in the normal population, at a measurable level but below the clinical diagnosis criteria^8^. For example, it has been shown that especially those suffering from positive symptoms including delusions have impairments in predicting the sensory feedback from own actions^9–12^. In healthy individuals, those having a high tendency of delusional thinking also show a similar deficit in predicting sensory feedback^13–16^. If delusions are a key factor contributing to the strong action binding in schizophrenia, we might also find a similar trend in the healthy population, i.e. individuals with a high tendency of delusional thinking may exhibit a strong action binding effect.

Delusional ideation can be assessed by established questionnaires, as we did in the current study using the PDI-21 questionnaire^17^. However, measuring action binding is not as straightforward as it seems to be. As introduced earlier, the action binding effect is the difference in action time perception between the operant condition and the baseline condition. When both conditions were tested in a mixed session (i.e. each trial was randomly assigned to either condition), Moore, et al.^18^ showed that the size of action binding in a given trial depends on the trial history (whether the trial followed a trial from the baseline condition or a trial from the operant condition). The study hinted that the size of action binding may be influenced by the testing order between the operant condition and the baseline condition when the conditions are tested separately, as did other studies^18–21^. Therefore, clarifying and eliminating the influence of testing order on the estimation of action binding is a necessary step before action binding can be meaningfully related to a psychological trait. To the best of our knowledge, the issue of testing order in action binding has not been formally investigated.

In the current study, we first evaluated the testing order effect in action binding using a novel testing method (see “Task, design, and procedure” and “Methodological consideration of action binding measurement” for details). After the confirmation of the testing order effect, measures were taken to alleviate the influence of testing order on the estimation of action binding before a correlation analysis was performed between action binding and the tendency of delusional thinking over participants. Indeed, participants with a higher tendency of delusional thinking were found to have a stronger action binding effect.

## Material and methods

### Participants

58 participants were recruited from a local participant pool (mean age = 27.3, *SD* = 9.7, 48 females). Written informed consent was obtained prior to the study, and monetary compensation was given after the study. The local ethics committee approved the study (Institute of Psychology, Faculty for Human Sciences, University of Würzburg; project number: GZ 2018-27). We followed the Declaration of Helsinki and the European data protection law (GDPR) in the course of experiments.

The correlation between the delusion score and action binding was 0.38 in a schizophrenia sample^7^. We assume a correlation coefficient of 0.3 among normal participants. According to G*Power^22^, a one-tailed Pearson’s correlation test (alpha = 0.05) should achieve a statistical power of 0.75 with a sample size of 58, and a statistical power of 0.65 with 45 (the actual sample size included in the correlation analysis).

### Task, design, and procedure

**Figure 1:**
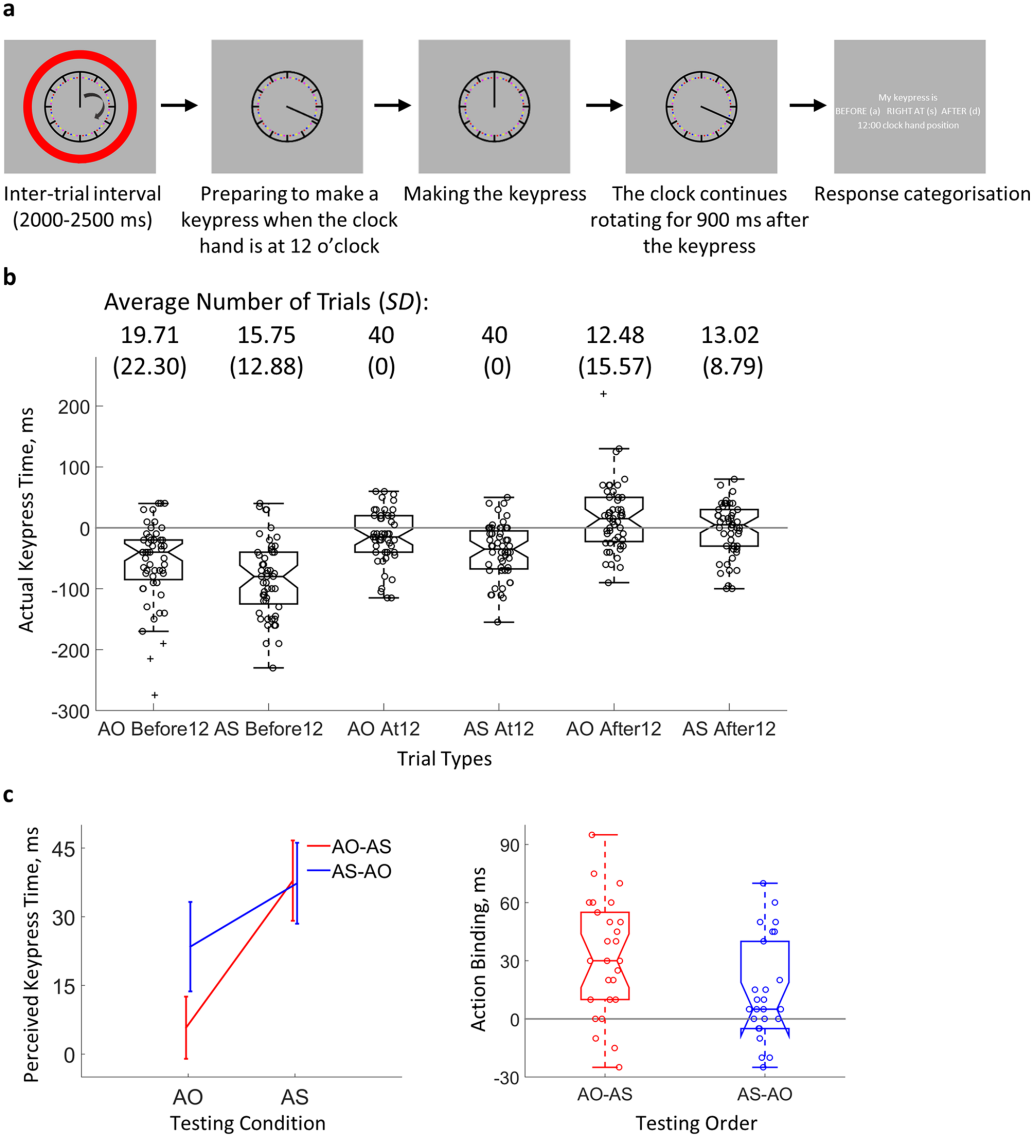
Trial structure and the testing order effect. (**a**) A schematic illustration of a trial. During the inter-trial interval, a red circle surrounds the rotating clock, and no responses are required. After the disappearance of the red circle, participants can make a keypress according to the instruction, i.e. try their best to press the key when the clock hand is at 12 o’clock position. After a response is made, the clock hand continues rotating for 900 ms before the participants are asked to evaluate whether the keypress is made before, at, or after 12 o’clock position. (**b**) The actual keypress time. The numbers on the top are the average number of trials (*SD* in brackets). Participants made more keypresses before the 12 o’clock position than after the 12 o’clock position (*t*(51) = 3.15, *p* = 2.71e−3, two-tailed, *dz* = 0.44). The grey line marks the horizontal 0 line. Each circle represents a participant. The central mark of the boxplot is the median, the edges of the box are the 25th and 75th percentiles, and the whiskers extend to the most extreme data points within 1.5 times the interquartile range. Data points outside 1.5 times the interquartile range are marked with crosses. (**c**) The average perceived keypress time in both testing order groups is shown on the left, and the individual action binding effect is shown on the right. In the boxplot, each circle represents a participant. Bars represent ± 1 standard error. *AO* action only, *AS* action sound.

The experiment was conducted in a lit room. Participants completed the task in front of a computer with a 50 cm viewing distance. During the testing, a clock hand rotated clockwise on the screen (Fig. 1a). A novel modification was made in the measurement of the subjective keypress time. Participants were required to make a brisk keypress when the clock hand was at the 12 o’clock position (instruction to participants: try your best to make a keypress when the clock hand is exactly at the 12 o’clock position) and indicate whether the keypress was really made at 12 o’clock after the response. A yes response would indicate that the subjective time of the keypress was when the clock hand was at the 12 o’clock position. This is an important difference to the commonly used method of making a spontaneous keypress and reporting the clock hand position after the keypress e.g.^1^. The novel modification brings at least 4 advantages as compared to the common method: (1) Visual attention is more focused. In the common method, participants’ attention needs to cover the whole area of the clock face along with the instantaneous position of the clock hand. In the modified method, participants only need to focus on the area around the 12 o’clock position. (2) Reduced requirement for memory load in performing the task. In the common method, participants need to remember the clock hand position at the moment of keypress and report it later, which is not required in the modified method. As a consequence, the modified task is also less demanding. (3) Improved precision of subjective timing measurement (see more in the discussion), which may result from the previous two points. (4) Better control of potential confounding variables. In the common method, participants freely decide when to make a keypress. Therefore, the clock hand position at the time of keypress (seldom reported in the literature) is not controlled. It is a matter of debate whether the reported time is biased due to, e.g. directions of eye movements or attentional shift, at different clock hand positions. Although the action binding effect is computed as a difference between operant and baseline conditions, any influence from the clock hand position may only be balanced out at a theoretical level. The problem is elegantly resolved in the modified method as only the 12 o’clock hand position is relevant for performing the task, thus leaving no chance for any influence of the clock hand position. A summary of the comparison between the two testing methods is provided in Table 1.

**Table 1:** A summary of the comparison between the common method and the current method. Please refer to the text for details.

Comparison points	Testing method	
	The common method	The current method
Visual attention	The whole clock face	Around the 12:00 position
Memory load	Low	Not required
Measurement precision	Around 80 ms (SD)	Around 40 ms (SD)
Clock hand position	Random (theoretically)	Controlled

There were two conditions. In the Action Sound (AS) condition (operant condition), the keypress was followed by a 250 ms delayed sound. In the Action Only (AO) condition (baseline condition), there was no auditory feedback following the keypress. Half of the participants completed AS first (AS-AO group), and the other half completed AO first (AO-AS group). Participants could take a self-paced break between conditions. Each trial started with an inter-trial period, during which the clock hand started the clockwise rotation always from the 12 o’clock position. The clock hand rotated 2 degrees per refresh frame, and the screen refresh rate was 100 Hz (i.e. the rotating hand had a period of 1800 ms). The inter-trial period was marked visually by a red circle surrounding the clock (indicating that no keypress should be made) and was randomly sampled between 2000 and 2500 ms. After the inter-trial period, the red circle disappeared, indicating that a response could be made. Participants then made a keypress with their right index finger on the key ‘K’ when the clock hand was close to the 12 o’clock position. A 250 ms delayed sound (1000 Hz; 50 ms long; 5 ms rise/fall envelop; comfortable volume level; the empirically measured sound delay was in the range of 252 to 254 ms) was played after the keypress, depending on the condition. The clock hand continued rotating for 900 ms after the keypress. Therefore, there was no visual feedback for the task performance. Immediately after the clock hand stopped rotating, an on-screen question was presented asking participants to indicate whether the keypress was before the 12 o’clock position (pressing the key ‘A’), right at the 12 o’clock position (pressing the key ‘S’), or after the 12 o’clock position (pressing the key ‘D’) with a left finger keypress, after which the next trial started immediately. A condition was completed when 40 trials with the response of pressing right at the 12 o’clock position were collected. In the end of the testing, participants completed the PDI-21 questionnaire^17^ (we used the German version; Lincoln, et al.^23^). The force value of each ‘K’ keypress was additionally recorded with a force sensing resistor but not analysed further here.

The Psychtoolbox-3 from Matlab (The MathWorks Inc., USA) was used for the task presentation^24^. The software Lab Streaming Layer was used for the data collection (https://github.com/sccn/labstreaminglayer).

### Data analysis

Two participants left the experiment before the testing finished (one in the AS-AO group and one in the AO-AS group). Their data were not considered. The physical keypress time point (t1), i.e. the registered keypress time stamp by the computer, and the time point when the clock hand was at the 12 o’clock position (t2) were extracted for each trial.

The main aim of this study was to test the hypothesis about a correlation between delusional ideation and action binding among healthy individuals. We first tested the action binding effect in the data. However, before performing the correlation analysis we needed to exclude the possible testing order effect, which could mask the correlation. We therefore secondly tested for the testing order effect, and in a third step, established a cut-off point after which the testing order effect was likely negligible. After that, the correlation analysis between delusional ideation and action binding was performed.

#### Action binding

To assess the action binding effect, only trials in which participants indicated that the keypress was made at the 12 o’clock position were considered. This is because the reported keypress time in those trials was known to be at the 12 o’clock position, whereas the reported keypress time in other trials (‘before 12’ or ‘after 12’) could not be accurately determined. For ‘at 12’ trials, the perceived keypress time in each trial was defined as the reported keypress time relative to the physical keypress time (i.e. t2–t1). For each participant, the median of the perceived keypress time in each condition was obtained (note that the median was used as the measure of central tendency for each condition at the participant level throughout the manuscript). Outlier participants were excluded based on the average perceived keypress time of AO and AS conditions using the MAD-median rule: let p be the individual average perceived keypress time and P be the set of perceived keypress time of all individuals. If |p – median(P)|× 0.6745 > 3 × MAD-median (the Median Absolute Deviation from the median), this individual is an outlier. Four outliers were identified. Therefore, the action binding effect analysis was based on the remaining 52 participants. For trials in which participants indicated that the keypress was made ‘before 12’ or ‘after 12’, the validity of responses was checked. The actual keypress time, i.e. the physical keypress time referenced to the time point when then clock hand was at the 12 o’clock position (t1–t2), was calculated for each trial. The actual keypress time should be earlier in trials responding that the keypress time was ‘before 12’ than in trials responding ‘after 12’ (Fig. 1b). Paired t-tests of the perceived keypress time between AS and AO conditions were performed to assess the action binding effect. A Cohen’s d was calculated to indicate the effect size. For Paired comparisons, $dz= \frac{t}{\sqrt{n}}$; For unpaired comparisons, $ds=t\sqrt{\frac{1}{{n}_{1}}+\frac{1}{{n}_{2}}}$, where t is the t-value, n, n_1_, and n_2_ are the sample sizes.

#### The testing order effect

An unpaired t-test was performed to compare the action binding effect (the perceived time in the AS condition minus the perceived time in the AO condition) between the AO–AS group and the AS–AO group for assessing the testing order effect. For each participant, a linear regression analysis using least squares was performed to assess the temporal development of perceived keypress time over trials separately for each testing condition. The regression slope was then compared against 0 at the group level separately for each testing condition in each testing order group. The influence of testing order on the development of perceived keypress time was evaluated by comparing the regression slope of a testing condition between the two testing order groups.

#### Establishing a cut-off point for the testing order effect

**Figure 2:**
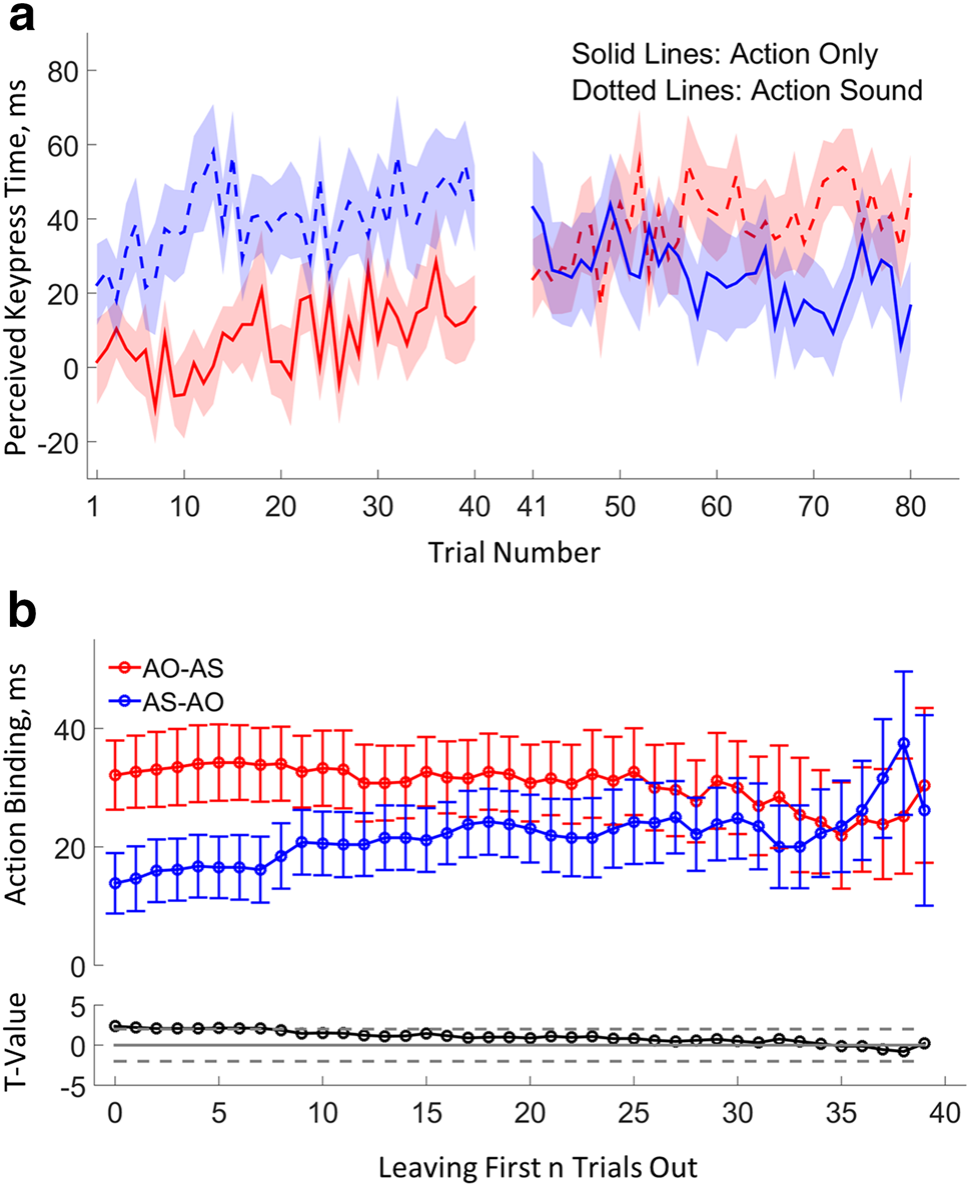
Results from the trial-by-trial analysis. (**a**) Trial-by-trial development of perceived keypress time in each condition. Bold lines show the average perceived keypress time of each trial over participants. The shading area represent ± 1 standard error. Note that only trials in which participants indicated that the keypress was made at 12 o’clock position are included here. Therefore, trial 13 is not really be the 13^th^ trials in a testing condition if participants made some presses ‘before 12’ or ‘after 12’ before trial 13. (**b**) The results from the leaving first n trials out analysis. The top row shows the average action binding effect in each testing order group with bars representing ± 1 standard error. The bottom row shows the t-value of the testing order effect comparison, with the solid grey line marking t-value of 0, and the two dotted grey lines marking the t-value threshold with a *p* value of 0.05 is obtained (two-tailed without correction for multiple comparisons). When n = 0 (x-axis), no trials are excluded. When n = 13, the first 13 trials in each condition are excluded.

The effect of testing order on action binding showed that the experience of an earlier tested condition influenced the timing perception in the later tested condition. This influence should get weaker over trials (strongest influence was expected for the first few trials in the later tested condition). To further explore this possibility, trials from the beginning of each condition were progressively left out before the testing order effect was evaluated. In the first step, the first trial of each condition was excluded and then the testing order effect was evaluated in the same way as if no trials were excluded (the mean and the standard error of the action binding effect in each testing order group, and the unpaired t-value comparing the two testing order groups were calculated; Fig. 2b). The procedure was repeated by excluding the first 2 trials, first 3 trials, until the first 39 trials (i.e. only the last trial was left in each condition). The cut-off point was chosen so that the testing order effect was not statistically detectable anymore and a sufficient number of trials were still left for estimating the action binding effect. For this reason, the first 10 trials from each condition were excluded to mitigate the influence of the testing order effect in the correlation analysis.

#### Correlation between delusional ideation and action binding

The PDI-21 questionnaire has 21 items covering various aspects of delusional thinking. For each item, participants could answer yes (1 point) or no (0 point). If yes was answered, participants were further asked to indicate the level of distress, preoccupation, and conviction towards the item on a 1–5 scale. A PDI overall score was calculated by adding up the points scored in all questions. The PDI overall score ranges from 0 to 336 (21 × (1 + 5 + 5 + 5)). The higher the score, the stronger the tendency of delusional thinking. Pearson’s correlation was computed between PDI overall score and action binding after possible outliers were excluded using the boxplot rule and the confidence interval of the correlation coefficient was obtained via bootstrapping^25^. In this case only, the individual t-value from the unpaired t-test of the perceived keypress time between AS and AO conditions was used to represent the strength of the individual action binding effect, since the t-value takes into account the variance^5^. Note that the t-value is equally sensitive to individual differences as Cohen’s d when the number of trials is the same among all individuals, which is the case in the current study. The PDI data from one participant was incomplete and six participants were identified as outliers. Therefore, the actual sample size in the correlation analysis was 45 (52-1-6).

## Results

### The validity of timing judgement

We first checked the validity of the response at the end of each trial (‘before 12’, ‘at 12’, or ‘after 12’). Indeed, the actual keypress time was earlier in ‘before 12’ trials (participants reporting that they pressed the key before the clock hand was at the 12 o’clock position) (mean = − 69.76 ms, *SD* = 55.11 ms) than in ‘at 12’ trials (mean = − 26.11 ms, *SD* = 41.42 ms; *t*(51) = 10.11, *p* = 4.44e−14, two-tailed, *dz* = 1.40), and earlier in ‘at 12’ trials than in ‘after 12’ trials (mean = 6.44 ms, *SD* = 43.45 ms; *t*(51) = 10.16, *p* = 3.75e-14, two-tailed, *dz* = 1.41), indicating the validity of the subjective report on the keypress time (Fig. 1b). The perceived keypress time in ‘before 12’ trials and ‘after 12’ trials could not be accurately determined. Nevertheless, if the perceived keypress time in ‘before 12’ trials and ‘after 12’ trials was calculated the same way as in ‘at 12’ trials, significant action binding can also be found in ‘before 12’ trials (*t*(51) = 2.83, *p* = 3.31e−3, one-tailed, *dz* = 0.39) and ‘after 12’ trials (*t*(51) = 2.90, *p* = 2.72e−3, one-tailed, *dz* = 0.40).

### The testing order affects action binding measurement

The testing order effect was explored with the aim of obtaining an accurate estimation of action binding for the planned correlation analysis. A clear action binding effect was found in the whole sample (*t*(51) = 5.68, *p* < 0.001, one-tailed, *d*_*z*_ = 0.79), i.e. the perceived keypress time was later in the AS condition (mean = 37.60 ms, 95% CI = [25.52 49.68]) than in the AO condition (mean = 14.62 ms, 95% CI = [2.82 26.41]), indicating a shift of the perceived keypress time towards the sound presentation time in the AS condition. Looking at the action binding effect separately for each testing order group (Fig. 1c), the AO-AS group showed a significant action binding effect (*t*(25) = 5.50, *p* < 0.001, one-tailed, *d*_*z*_ = 1.08; AO: mean = 5.77 ms, *95% CI* = [− 7.53 19.07]; AS: mean = 37.88 ms, *95% CI* = [20.70 55.07]). A significant action binding effect was also found in the AS-AO group (*t*(25) = 2.71, *p* = 0.006, one-tailed, *d*_*z*_ = 0.53; AO: mean = 23.46 ms, *95% CI* = [4.32 42.60]; AS: mean = 37.31 ms, *95% CI* = [19.99 54.63]). Importantly, the action binding effect was significantly larger in the AO-AS group than in the AS-AO group (*t*(50) = 2.36, *p* = 0.022, two-tailed, *d*_*s*_ = 0.65). To check the influence of the testing order on a single condition, we compared the same testing condition between the two testing orders. The absolute change in perceived keypress time due to testing order was 17.69 ms for the AO condition and only 0.58 ms for the AS condition, although the absolute change was not statistically different from 0 for either condition (*p* > 0.14, two-tailed). We next investigated the trial-by-trial development of perceived keypress time to gain further insights into the testing order effect.

### Trial-by-trial analysis of action binding

A linear regression analysis was performed to explore the development of perceived keypress time over trials. The results showed that the perceived keypress time was not stable over trials (Fig. 2a). In the AO condition, the AO–AS group showed an increase of perceived keypress time over trials, as indicated by a positive regression slope (*t*(25) = 2.88, *p* = 0.008, two-tailed, *d*_*z*_ = 0.56), whereas the AS–AO group showed a decrease pattern (*t*(25) =  − 2.74, *p* = 0.011, two-tailed, *d*_*z*_ = − 0.54). Critically, the regression slope in the AO condition was significantly higher in the AO–AS group than in the AS–AO group (*t*(50) = 3.96, *p* < 0.001, two-tailed, *d*_*s*_ = 1.10), which is consistent with the idea that mainly the AO condition received strong influence from the testing order. In the AS condition, both testing groups showed weak signs of an increase in the perceived keypress time over trials (AO–AS group: *t*(25) = 2.16, *p* = 0.040, two-tailed, *d*_*z*_ = 0.42; AS–AO group: *t*(25) = 1.77, *p* = 0.090, two-tailed, *d*_*z*_ = 0.35), with no significant difference in the regression slope being found between the two groups (*t*(50) =  − 0.17, *p* = 0.863, two-tailed, *d*_*s*_ = − 0.05).

The perceived keypress time seemed to stabilise after a few trials of rapid changes in the beginning phase of a testing condition, suggesting that there may be a genuine size of action binding which is independent of the testing order effect. Through progressively leaving out trials from the beginning of each testing condition before the action binding effect was calculated and compared between the two testing order groups, the results showed that the testing order effect started to, and consistently thereafter, fall above the statistical significance threshold (*p* = 0.05) when the first 8 trials from each condition were omitted in the action binding calculation (Fig. 2b).

### A positive correlation between delusional thinking and action binding

**Figure 3:**
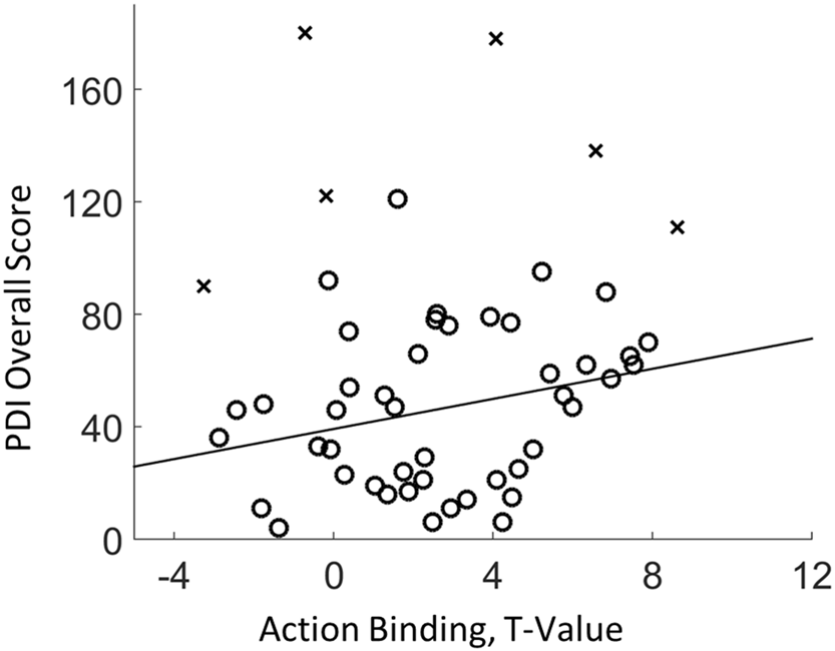
Scatter plot between PDI overall score and action binding. A high PDI overall score is associated with a strong action binding effect. Each circle represents a participant. Outliers are marked by crosses. The solid line shows the linear fitting between the two variables.

The strength of action binding for each participant was then represented using the t-value from the unpaired t-test of perceived keypress time between the AS and AO conditions, with the first 10 trials from each condition left out. Consistent with our prediction, a positive correlation between PDI overall score and action binding was found across participants (Pearson’s *r* = 0.27, *p* = 0.039, one-tailed, *95% CI* = [0.03 0.49], bootstrap). The positive correlation indicates that participants with a high PDI overall score, i.e. high tendency towards delusional thinking, showed a strong action binding effect (Fig. 3).

## Discussion

We showed that a high tendency of delusional thinking was associated with a strong action binding effect among healthy individuals, with the estimation of individual action binding controlled for the testing order effect (i.e. action binding is larger when the AO condition is tested first than when the AS condition is tested first). The current study contributes to our understanding of the strong action binding effect in schizophrenia and also has practical implications for the measurement of action binding.

### Strong action binding in schizophrenia

It has been suggested that action binding has two components: a predictive component and a retrospective component^7,19^. Moore and Haggard^19^ showed that when a keypress only triggered a tone with 75% probability, a very strong action binding could be found in the 25% trials in which no tone was played after a keypress (i.e. the predictive component). The authors took this as evidence that the prediction of sensory feedback from own actions contributes towards action binding. The retrospective component is formed by inferring the action time from sensory evidence after the action has been performed, which is implemented as a process of cue integration or multisensory information integration^3,5,26–28^. In particular, Voss, et al.^7^ showed that it was the predictive component that had a negative correlation with positive symptoms in schizophrenia, which is consistent with other reports of a prediction deficit in schizophrenia^9–12^. If the predictive component does not contribute to the action binding effect observed in schizophrenia, the strong action binding effect in schizophrenia can only be caused by a strong retrospective component^29^. In the current data, a positive correlation between the tendency of delusional thinking and action binding was found, which would suggest that our measure of action binding largely reflects the retrospective component.

Why is there a positive correlation between the tendency of delusional thinking and the retrospective component of action binding? As we discussed earlier, the retrospective component is implemented as a process of cue integration. When participants judged the keypress time, both the keypress itself (e.g. somatosensory feedback) and the self-generated sound served as cues for the time estimation^3,5^. A strong tendency of delusion thinking should lead to more reliance on the timing information from the sound. This is because of an effect called sensory attenuation (i.e. self-generated sound leads to attenuated brain responses as compared to an externally generated sound). Sensory attenuation is generally believed to be caused by a prediction of sensory feedback from own actions^30–33^. The delusion symptom in schizophrenia has been shown to be related to a reduction in sensory attenuation^34,35^. In healthy individuals, a high tendency of delusional thinking is also shown to be associated with a weakened sensory attenuation, which may be mediated through an impaired prediction^13–15^. With weakened sensory attenuation, the effectiveness of the self-generated sound in binding the action time towards the sound can probably be enhanced, i.e. a strong action binding. This explanation connects the predictive component and the retrospective component via the impact of prediction on sensory processing, which also provides a grounding point for prediction to act on action time perception (cf. Waszak et al.^36^). Further investigation is needed to firmly establish the hypothesis.

### Methodological consideration of action binding measurement

The current study employed a modified method to evaluate the subjective timing of a keypress. Specifically, participants were asked to make a keypress when the rotating clock hand was at the 12 o’clock position and indicate if the keypress was really made at 12 at the end of the trial. This is in contrast to the common method of asking participants to make a spontaneous keypress and report the position of the clock hand at the moment of the keypress later^1^. The validity of the modified method is indicated by the successful replication of the action binding effect. The size of action binding is also comparable between the current study (23 ms; n = 52) and our previous study using the common method (25 ms; n = 42)^5^. The advantages of the modified method have been outlined in Task, design, and procedure. Here we would like to unfold the major advantage of the modified method, i.e. a substantial improvement in the precision of the subjective keypress time measurement. The precision of measurement can be quantified by the standard deviation. In our case, it would be the standard deviation of the perceived keypress time in each condition. The average standard deviation of the perceived keypress time was 40.94 ms (*SD* = 14.39) in the AO condition and 41.23 (*SD* = 13.47) in the AS condition. Note that this is the result without any trial exclusion. For comparison, the standard deviation obtained with the common method is at least 80 ms, quite often over 100 ms (for a list of action binding studies, see Tanaka, et al.^37^). The improvement of the measurement precision is particularly important for a deeper understanding of the effect investigated here. The cue integration account of action binding is gaining substantial support from empirical studies^3,5^. The precision of timing judgement is a crucial component in the cognitive modelling of the cue integration account. However, a recent model based on the precision data obtained from the common method failed to replicate a robust feature in intentional binding studies, i.e. the size of action binding is much smaller than the size of outcome binding^38^. Outcome binding is the effect that the outcome of an action is temporally shifted towards the action^1,39^. Our modified method may contribute to the development of a more realistic model of intentional binding (outcome binding may also be measured using the modified method). Furthermore, the modified method may be improved in the way that instead of responding ‘before 12’ or ‘after 12’, participants could report the exact position of the clock hand in these trials. This should not bring any negative effect to the precision of the measurement as the keypress should always be made at some point close to the 12 o’clock position. There is, however, one concern over the improvement in the measurement precision. The improved measurement precision may be contributed by an extremely precise timing in motor execution, which can lead to a bias in the sampling of the perceived keypress time (i.e. only a subgroup of the perceived keypress time being sample can also lead to a reduction in standard deviation). Although there are quite some trials in which participants responded with ‘before 12’ or ‘after 12’ (Fig. 1b), suggesting that the above concern may not apply to the current study, further studies are needed to fully clarify this issue.

The testing order effect, i.e. the AO-AS group (starting with the AO condition) showed a much larger action effect than the AS-AO group (starting with the AS condition), reflects an asymmetry in the interaction between AO and AS conditions. An increase of perceived keypress time over trials seems to be a common feature in both AO and AS conditions when there is no influence from a previous testing condition. However, when participants were tested in the AO condition directly after being tested in the AS condition (AS-AO group), a decrease of perceived keypress time over trials was found. This indicates an influence of the AS condition towards the AO condition. The relatively ‘late’ perceived keypress time in the preceding AS condition was still present in the first few trials of the AO condition. With the testing moving on, the perceived time in the AO condition gradually decoupled from the influence of the preceding AS condition and returned to its own range of relatively ‘early’ perceived keypress time, i.e. a decrease of the perceived keypress time over trials. However, a similar influence from the AO condition to AS condition was not found in the AO-AS group. If the first few trials of the AS condition carried some influence from the preceding AO condition, a steeper regression slope in the AS condition should be found in the AO-AS condition than in the AS-AO condition, which is not the case.

The testing order effect has practical implications for action binding measurement. It shows that a canonical counterbalancing of the testing order between participants is not a good strategy, especially in the case when an accurate estimation of the individual action binding effect is important. To mitigate the influence of testing order on the estimation of action binding, we explored the possibility of excluding a few trials from the start of each testing condition, which may carry very strong influence from a preceding testing condition. Results showed that the testing order effect was not statistically significant if the first 8 trials were excluded. This is a clear message for action binding studies in which an accurate estimation of the effect size is important. However, there are a few caveats that should be kept in mind. First, the assumption of the existence of a veridical action binding effect may not be true. It is not clear if the perceived keypress time undergoes constant development if more testing trials in each condition is included, although the development pattern over 40 trials included in the current study showed that the perceived keypress time was relatively stable after around 10 trials (Fig. 2a). Second, the absence of statistical significance in the testing order effect does not necessarily mean that the testing order effect is completely eliminated. A numerical difference of about 10 ms in the testing order effect was still present when the first 10 trials were excluded (Fig. 2b). Nevertheless, the testing order constitutes an important factor in explaining the individual difference in the action binding effect and should be considered or controlled for when explaining the action binding effect in relevant studies^37,40^. In this sense, it may also be an interesting idea to investigate if a similar testing order effect is present in outcome binding.

## Conclusion

There is a positive correlation between the tendency of delusional thinking and action binding in healthy individuals. The positive correlation may suggest an alternative explanation of prediction in action binding, i.e. it reduces action binding through attenuating the effectiveness of the binding effect from reafferent inputs (sound). The testing order effect of action binding argues that the testing order should be controlled for and cannot be simply counterbalanced out in the standard action binding study design.

## Data Availability

The data and Matlab analysis code have been deposited in Figshare for free access (https://doi.org/10.6084/m9.figshare.15128646).

## References

[1] Haggard P, Clark S, Kalogeras J (2002). Voluntary action and conscious awareness. Nat. Neurosci..

[2] Caspar EA, Christensen JF, Cleeremans A, Haggard P (2016). Coercion changes the sense of agency in the human brain. Curr. Biol..

[3] Wolpe N, Haggard P, Siebner HR, Rowe JB (2013). Cue integration and the perception of action in intentional binding. Exp. Brain Res..

[4] Buehner MJ, Humphreys GR (2009). Causal binding of actions to their effects. Psychol. Sci..

[5] Cao L, Steinborn M, Kunde W, Haendel B (2020). Action force modulates action binding: Evidence for a multisensory information integration explanation. Exp. Brain Res..

[6] Haggard P, Martin F, Taylor-Clarke M, Jeannerod M, Franck N (2003). Awareness of action in schizophrenia. NeuroReport.

[7] Voss M (2010). Altered awareness of action in schizophrenia: A specific deficit in predicting action consequences. Brain.

[8] van Os J, Linscott RJ, Myin-Germeys I, Delespaul P, Krabbendam L (2009). A systematic review and meta-analysis of the psychosis continuum: Evidence for a psychosis proneness-persistence-impairment model of psychotic disorder. Psychol. Med..

[9] Synofzik M, Thier P, Leube DT, Schlotterbeck P, Lindner A (2010). Misattributions of agency in schizophrenia are based on imprecise predictions about the sensory consequences of one's actions. Brain.

[10] Shergill SS, Samson G, Bays PM, Frith CD, Wolpert DM (2005). Evidence for sensory prediction deficits in schizophrenia. Am. J. Psychiat..

[11] Ford JM (2001). Cortical responsiveness during inner speech in schizophrenia: An event-related potential study. Am. J. Psychiat..

[12] Shergill SS (2014). Functional magnetic resonance imaging of impaired sensory prediction in schizophrenia. JAMA Psychiat..

[13] Oestreich LKL (2015). Subnormal sensory attenuation to self-generated speech in schizotypy: Electrophysiological evidence for a 'continuum of psychosis'. Int. J. Psychophysiol..

[14] Teufel C, Kingdon A, Ingram JN, Wolpert DM, Fletcher PC (2010). Deficits in sensory prediction are related to delusional ideation in healthy individuals. Neuropsychologia.

[15] Cao L, Gross J (2015). Cultural differences in perceiving sounds generated by others: Self matters. Front. Psychol..

[16] Malassis R, Del Cul A, Collins T (2015). Corollary discharge failure in an oculomotor task is related to delusional ideation in healthy individuals. PLoS ONE.

[17] Peters E, Joseph S, Day S, Garety P (2004). Measuring delusional ideation: The 21-item Peters et al. delusions inventory (PDI). Schizophrenia Bull..

[18] Moore JW, Middleton D, Haggard P, Fletcher PC (2012). Exploring implicit and explicit aspects of sense of agency. Conscious Cogn..

[19] Moore JW, Haggard P (2008). Awareness of action: Inference and prediction. Conscious Cogn..

[20] Walsh E, Haggard P (2013). Action, prediction, and temporal awareness. Acta Psychol..

[21] Matute H, Cubillas CP, Garaizar P (2017). Learning to infer the time of our actions and decisions from their consequences. Conscious Cogn..

[22] Faul F, Erdfelder E, Lang AG, Buchner A (2007). G*Power 3: A flexible statistical power analysis program for the social, behavioral, and biomedical sciences. Behav. Res. Methods.

[23] Lincoln TM, Keller E, Rief W (2009). An assessment of delusional ideation and hallucination in the general population: German adaptations of the Peters et al. Delusions Inventory (PDI) and the Launay-Slade Hallucination Scale (LSHS-R). Diagnostica.

[24] Kleiner, M., Brainard, D. & Pelli, D. What's new in Psychtoolbox-3? (2007).

[25] Pernet CR, Wilcox R, Rousselet GA (2013). Robust correlation analyses: False positive and power validation using a new open source matlab toolbox. Front. Psychol..

[26] Lau HC, Rogers RD, Passingham RE (2007). Manipulating the experienced onset of intention after action execution. J. Cognit. Neurosci..

[27] Moore JW, Fletcher PC (2012). Sense of agency in health and disease: A review of cue integration approaches. Conscious Cogn..

[28] Kirsch W, Kunde W, Herbort O (2019). Intentional binding is unrelated to action intention. J. Exp. Psychol. Human.

[29] Graham-Schmidt KT, Martin-Iverson MT, Holmes NP, Waters FAV (2016). When one's sense of agency goes wrong: Absent modulation of time perception by voluntary actions and reduction of perceived length of intervals in passivity symptoms in schizophrenia. Conscious Cogn..

[30] Cao L, Veniero D, Thut G, Gross J (2017). Role of the cerebellum in adaptation to delayed action effects. Curr. Biol..

[31] Schroger E, Marzecova A, SanMiguel I (2015). Attention and prediction in human audition: A lesson from cognitive psychophysiology. Eur. J. Neurosci..

[32] Schafer EWP, Marcus MM (1973). Self-stimulation alters human sensory brain responses. Science.

[33] Martikainen MH, Kaneko K, Hari R (2005). Suppressed responses to self-triggered sounds in the human auditory cortex. Cereb. Cortex.

[34] Lindner A, Thier P, Kircher TTJ, Haarmeier T, Leube DT (2005). Disorders of agency in schizophrenia correlate with an inability to compensate for the sensory consequences of actions. Cur. Biol..

[35] Blakemore SJ, Smith J, Steel R, Johnstone EC, Frith CD (2000). The perception of self-produced sensory stimuli in patients with auditory hallucinations and passivity experiences: Evidence for a breakdown in self-monitoring. Psychol. Med..

[36] Waszak F, Cardoso-Leite P, Hughes G (2012). Action effect anticipation: Neurophysiological basis and functional consequences. Neurosci. Biobehav. R.

[37] Tanaka T, Matsumoto T, Hayashi S, Takagi S, Kawabata H (2019). What makes action and outcome temporally close to each other: A systematic review and meta-analysis of temporal binding. Timing Time Percept..

[38] Legaspi R, Toyoizumi T (2019). A Bayesian psychophysics model of sense of agency. Nat. Commun..

[39] Cravo AM, Claessens PME, Baldo MVC (2011). The relation between action, predictability and temporal contiguity in temporal binding. Acta Psychol..

[40] Moore JW, Obhi SS (2012). Intentional binding and the sense of agency: A review. Conscious Cogn..

