# Author Correction: Delusional thinking and action binding in healthy individuals

**DOI:** 10.1038/s41598-021-00196-8

**Published:** 2021-10-14

**Authors:** Liyu Cao, Michael B. Steinborn, Barbara F. Haendel

**Affiliations:** 1 Department of Psychology and Behavioural Sciences, Zhejiang University, Tianmushan Road 148, Hangzhou, 310007 China; 2 Department of Psychology (III), Julius-Maximilians-Universität Würzburg, 97070 Würzburg, Germany

Correction to: *Scientific Reports* 10.1038/s41598-021-97977-y, published online 23 September 2021

The original version of this Article contained errors in Table 1, where the measurement precision values for “The common method” and “The current method” were inadvertently switched. The incorrect and correct values appear below.

Incorrect:

**Table:** 

Comparison points	Testing method	
	The common method	The current method
Measurement precision	Around 40 ms (SD)	Around 80 ms (SD)

Correct:

**Table:** 

Comparison points	Testing method	
	The common method	The current method
Measurement precision	Around 80 ms (SD)	Around 40 ms (SD)

The original Article has been corrected.

